# An Unsuspected Finding of t(9;22): A Rare Case of Philadelphia Chromosome-Positive B-Lymphoblastic Lymphoma

**DOI:** 10.1155/2017/2413587

**Published:** 2017-09-18

**Authors:** Prajwal Boddu, C. Cameron Yin, Rashmi Kanagal-Shamanna, Guillin Tang, Beenu Thakral, Tapan Kadia, Marina Konopleva, Elias Jabbour, Nitin Jain

**Affiliations:** ^1^Department of Leukemia, UT MD Anderson Cancer Center, Houston, TX, USA; ^2^Department of Hematopathology, UT MD Anderson Cancer Center, Houston, TX, USA

## Abstract

While rare, cases of isolated extramedullary disease of B-cell Lymphoblastic Lymphoma (B-LBL) without morphologic bone marrow involvement have been described. In this report, we illustrate the case of an elderly gentleman who presented with isolated testicular and vertebral LBL involvement but had no morphologic bone marrow involvement. The initial plan of treatment was to treat along the lines of Philadelphia negative B-ALL/LBL. During this time, fluorescence in situ hybridization (FISH) and PCR testing for BCR-ABL1 rearrangements were being performed on the marrow specimens as a part of routine diagnostic workup. While the FISH returned negative, PCR testing unexpectedly detected BCR-ABL1 fusion transcripts at a low level of 0.48%. Given their presence, we performed FISH for BCR/ABL1 rearrangement in both testicular and L5 vertebral specimens which were 80–90% positive. He subsequently received rituximab, hyper-CVAD, and dasatinib, along with prophylactic intrathecal prophylactic chemotherapy. The patient achieved a prolonged remission but eventually relapsed, 4 years later. Had it not been for this fortuitous discovery, the patient would not have been treated with tyrosine kinase inhibitors. We emphasize that FISH and PCR testing for BCR-ABL1 rearrangement are integral to arriving at an accurate diagnosis and should be routinely tested on B-LBL biopsy specimens.

## 1. Introduction

Acute lymphoblastic leukemia/lymphoma (ALL/LBL) is a malignant neoplasm characterized by rapid proliferation of immature lymphoblasts in the bone marrow and extramedullary tissues. An arbitrary distinction between leukemia and lymphoma in the ALL/LBL disease spectrum is made based on the percentage of bone marrow blasts with more than 25% designated as leukemia [1]. LBL is a disease primarily occurring in children and young adults but has also been reported across all age groups [2]. B-LBL has a much lower incidence (approximately 10% of all LBL), predominates in older age population, and is less likely to present with bone marrow involvement compared with its T-cell counterpart [2, 3].

The presence of* BCR-ABL1* fusion defines Philadelphia chromosome-positive ALL (Ph+ ALL). Ph+ ALL constitutes only 2–5% of childhood cases but is the most common cytogenetic abnormality occurring in up to 30–40% of adult ALL patients [4, 5]. The diagnosis has major therapeutic implications with ABL1 tyrosine kinase inhibitors forming a valuable component of the treatment strategy and positively influencing clinical outcome in an otherwise high-risk subset of ALL [6].

Extramedullary tissue involvement typically occurs during relapse or disease progression and it is uncommon to find isolated extramedullary disease without evidence of bone marrow involvement. Ph+ LBL has only rarely been reported in the literature but may be potentially underrecognized due to inadequate testing of the tumor samples [7]. We report a patient with Ph+ B-LBL. The diagnosis was based on clues obtained from molecular testing performed on his bone marrow. This case illustrates the necessity and immense value of performing adequate cytogenetic and molecular analyses to help arrive at an accurate diagnosis.

## 2. Case Report

**Figure 1:**
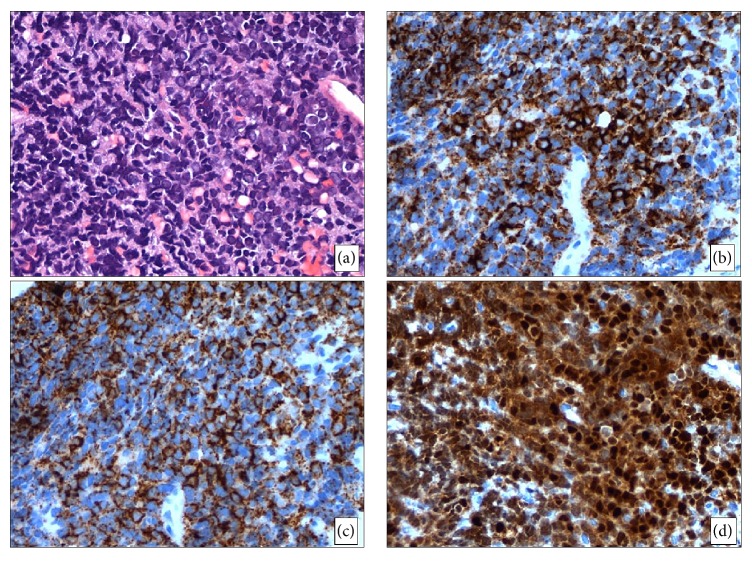
Morphologic features of the L5 lesion. (a) The core needle biopsy specimen is extensively replaced with atypical lymphoid infiltrate with predominantly medium-sized cells fine chromatin, occasional distinct nucleoli, and scant cytoplasm (H&E, 400x). (b–d) Immunohistochemical stains show that the neoplastic cells are positive for CD19 ((b), 400x), CD20 ((c), 400x), and TdT ((d), 400x).

**Figure 2:**
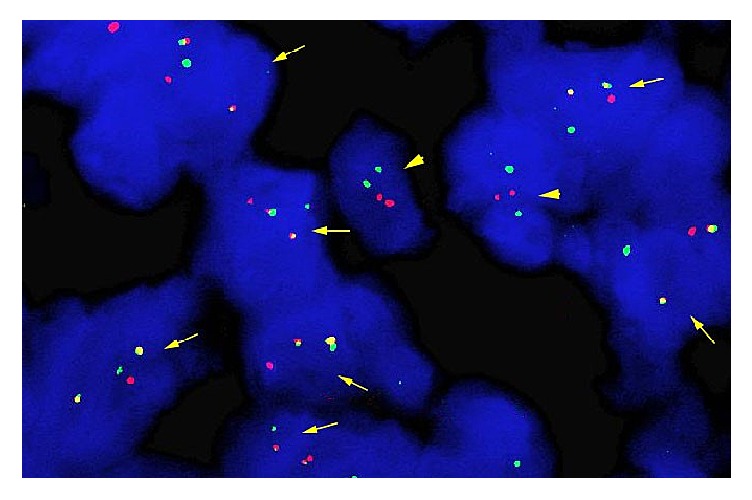
Fluorescence in situ hybridization analysis with a LSI BCR/ABL1 ES probes (Abbott Molecular, Inc.) on a formalin-fixed paraffin-embedded L5 vertebral body biopsy. Cells with a signal pattern of 1 red, 1 green, and 2 yellow (fusion signal) are positive for BCR/ABL1 rearrangement (marked by arrow); cells with 2 red and 2 green are normal (marked by arrow head).

**Figure 3:**
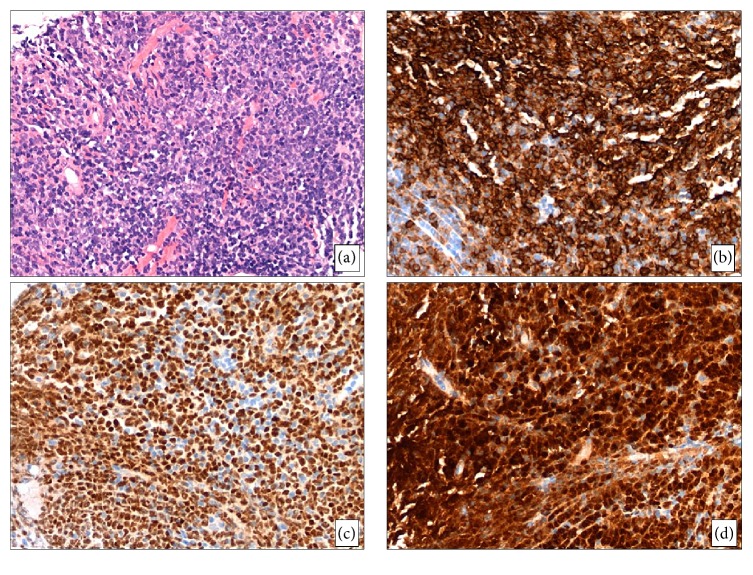
Morphologic features of the soft tissue lesion upon relapse. (a) The core needle biopsy specimen is extensively replaced with atypical lymphoid infiltrate with predominantly medium-sized cells with fine chromatin, occasional distinct nucleoli, and scant cytoplasm (H&E, 200x). (b–d) Immunohistochemical stains show that the neoplastic cells are positive for CD20 ((b), 200x), PAX-5 ((c), 200x), and TdT ((d), 200x).

A 77-year-old gentleman first presented to our institution four years ago for a second opinion and treatment options. His history dated back three months prior to presentation when he began to notice calf pain after biking. This was followed, a month later, by a mechanical fall after which he started experiencing constant mild back pain. During this time, he also noticed that his right testicle was enlarged. The testicular enlargement did not respond to antibiotics and he subsequently underwent an ultrasound which showed solid masses in both testes. PET scan demonstrated increased metabolic activity in the right testicle (SUV 6), a portion of the left testicle (SUV 5), and a flare in a 6 mm retroperitoneal lymph node (SUV 4). There was also increased metabolic activity observed in the lytic lesions involving the left pubic symphysis (SUV of 7.2) and L5 vertebral body (SUV 6.2). The patient underwent a bilateral orchiectomy and, soon after, presented to our institution. The pathology specimen was reviewed and showed lymphomatous involvement with sheets of medium-sized cells with blastoid morphology characterized by fine chromatin, occasional distinct nucleoli, and scant cytoplasm. Immunohistochemical stains revealed the neoplastic cells to be positive for CD10, CD34, CD43, BCL-2, and TdT and negative for CD3, CD5, and CD20. Flow cytometric immunophenotypic analysis showed an aberrant population of lymphoblasts that were positive for CD10, CD19, CD34, HLA-DR, and dim CD4 and negative for surface immunoglobulin light chains, consistent with B-LBL. A bone marrow biopsy performed at our institution showed only 2% blasts with no morphologic support for acute leukemia. Flow cytometry analysis of the bone marrow aspirate specimen revealed a very small population of B-lymphoblasts (0.006%), consistent with minimal disseminated disease (MDD) by B-ALL. Cytogenetic analysis performed on the bone marrow showed a normal male diploid karyotype, 46, XY[20]. Fluorescence in situ hybridization (FISH) using a probe specific for* BCR-ABL1* rearrangement was negative. However, real-time PCR testing for* BCR-ABL1* rearrangement, a part of routine panel testing at our institution, detected a low-level e1a2* BCR-ABL1* fusion transcript coding for the 190 kDa BCR-ABL1 protein (*BCR-ABL1/ABL1*, 0.48%). We subsequently performed a core needle biopsy of the patient's L5 vertebral lesion which was also consistent with B-LBL (Figure 1). Given the presence of low-level* BCR-ABL1* fusion in the bone marrow detected by PCR, we performed FISH analysis for BCR/ABL1 rearrangement in both the testicular biopsy and the L5 vertebral biopsy, which showed that 90% (testes) and 80% (L5 vertebral body) were positive for BCR/ABL1 rearrangement (Figure 2). Meanwhile, PCR performed on a peripheral blood sample also showed a low-level of* BCR-ABL1* gene rearrangement (1.46%). Based on these findings, the patient was diagnosed to have Ph+ B-LBL. He received rituximab, hyper-CVAD chemotherapy, and dasatinib, along with intrathecal prophylactic chemotherapy. He achieved a complete remission, documented by repeat marrow and PET imaging. He developed pleural effusion and fever with dasatinib, and thus dasatinib was switched to imatinib, and later nilotinib (for possible better antileukemia activity). Repeat PCR on bone marrow and peripheral blood samples showed no evidence of* BCR-ABL1* gene rearrangement. However, a routine yearly PET scan performed 4 years after the initial diagnosis showed a new soft tissue mass below the right kidney. A biopsy revealed relapsed B-LBL (Figure 3). Therapy with blinatumomab and ponatinib was planned. However, the patient developed aspiration pneumonia and died before therapy could be instituted.

## 3. Discussion

Testicular involvement by lymphoma is uncommon occurring in only 1-2% of extranodal lymphomas [8]. Primary testicular B-LBL is an infrequently reported entity and constitutes a very small percentage of testicular lymphomas [9, 10]. There was no morphologic evidence to suggest leukemia involvement with only 2% blasts on initial bone marrow analysis. We did not entertain the possibility of Ph+ B-LBL until the detection of low-level* BCR-ABL1* fusion on our routine bone marrow real-time PCR testing. Based on this finding, we assessed for the presence of* BCR-ABL1* fusion by FISH in his testicular and vertebral body biopsy samples which returned positive (Figure 2). This allowed us to make a diagnosis of Ph+ B-LBL and treat him with hyper-CVAD and a tyrosine kinase inhibitor (dasatinib). We would not have reached this diagnosis if we had not routinely performed real-time PCR (sensitivity 0.001%) [11]. Additional clues to Ph positivity may be obtained from flow cytometry. Although CD25 was not analyzed on the testis biopsy sample, it was tested for and returned positive in the bone marrow sample. CD25 is highly expressed in patients with BCR-ABL fusions [12]. Additionally, CD13 and CD33 tend to be expressed with higher intensity in Ph+ ALL [13].

Ph+ B-LBL has only rarely been reported in the literature. Zhu et al. reported a patient with testicular B-LBL in whom the presence of Ph chromosome was not detected until 8 months after initial diagnosis when he returned with a relapse [7]. It is conceivable that this may have had a major impact on the eventual outcome. Early and accurate diagnosis is imperative as best possible outcomes are obtained when tyrosine kinase inhibitors are instituted early in treatment along with the first cycle of induction therapy [14, 15]. It is noteworthy to mention that the testis, like central nervous system, provides a sanctuary site for cancer cells and its involvement predicts for a high risk of systemic and nervous system relapse [16, 17]. Despite the much more comprehensive treatment which included bilateral orchiectomy, rituximab, hyper-CVAD, and dasatinib with intrathecal prophylaxis, our patient eventually relapsed and died, approximately 4 years after the initial diagnosis.

In conclusion, we describe a rare case of Ph+ B-LBL. We recommend routine testing of B-LBL (and T-LBL) biopsy specimens for the presence of Philadelphia chromosome, preferably by both FISH and PCR.
